# Effects of a Gentle, Self-Administered Stimulation of Perineal Skin for Nocturia in Elderly Women: A Randomized, Placebo-Controlled, Double-Blind Crossover Trial

**DOI:** 10.1371/journal.pone.0151726

**Published:** 2016-03-22

**Authors:** Kaori Iimura, Nobuhiro Watanabe, Koichi Masunaga, Shogo Miyazaki, Harumi Hotta, Hunkyung Kim, Tatsuya Hisajima, Hidenori Takahashi, Yutaka Kasuya

**Affiliations:** 1 Graduate School of Health Science, Teikyo Heisei University, Tokyo, Japan; 2 Department of Autonomic Neuroscience, Tokyo Metropolitan Institute of Gerontology, Tokyo, Japan; 3 Department of Urology, Tokyo Metropolitan Geriatric Hospital, Tokyo, Japan; 4 Department of Acupuncture and Moxibustion, Faculty of Health Care, Teikyo Heisei University, Tokyo, Japan; 5 Department of Promotion of Prevention of Musculoskeletal Aging, Tokyo Metropolitan Institute of Gerontology, Tokyo, Japan; Carolina Urologic Research Center, UNITED STATES

## Abstract

**Background:**

Somatic afferent nerve stimuli are used for treating an overactive bladder (OAB), a major cause of nocturia in the elderly. Clinical evidence for this treatment is insufficient because of the lack of appropriate control stimuli. Recent studies on anesthetized animals show that gentle stimuli applied to perineal skin with a roller could inhibit micturition contractions depending on the roller’s surface material. We examined the efficacy of gentle skin stimuli for treating nocturia.

**Methods:**

The study was a cross-over, placebo-controlled, double-blind randomized clinical study using two rollers with different effects on micturition contractions. Participants were elderly women (79–89 years) with nocturia. Active (soft elastomer roller) or placebo (hard polystyrene roller) stimuli were applied to perineal skin by participants for 1 min at bedtime. A 3-day baseline assessment period was followed by 3-day stimulation and 4-day resting periods, after which the participants were subjected to other stimuli for another 3 days. The primary outcome was change in the frequency of nighttime urination, for which charts were maintained during each 3-day period.

**Results:**

Twenty-four participants were randomized, of which 22 completed all study protocols. One participant discontinued treatment because of an adverse event (abdominal discomfort). In participants with OAB (n = 9), change from baseline in the mean frequency of urination per night during the active stimuli period (mean ± standard deviation, −0.74 ± 0.7 times) was significantly greater than that during placebo stimuli periods (−0.15 ± 0.8 times [*p* < 0.05]). In contrast, this difference was not observed in participants without OAB (n = 13).

**Conclusions:**

These results suggest that gentle perineal stimulation with an elastomer roller is effective for treating OAB-associated nocturia in elderly women. Here the limitation was a study period too short to assess changes in the quality of sleep and life.

**Trial Registration:**

UMIN Clinical Trial Registry (CTR) UMIN000015809

## Introduction

Many elderly people have bladder problems such as frequent urination, incontinence, or nocturia [1]. Nocturia is a condition, in which the individual has to wake one or more times at night to void [2]. The prevalence of nocturia increases with age in both men and women and up to 60% of the elderly (≥70 years) void ≥2 times per night [3]. Nocturia should be appropriately managed, since it has been reported that nocturia not only decreases the quality of life (QOL) [4, 5], but also increases fall-related fracture risks and mortality rates [6]. One of the major causes of nocturia in the elderly is an overactive bladder (OAB). The most common treatment for OAB-associated nocturia is pharmacological treatment such as anticholinergic therapy. However, in a systematic review, its clinical efficacy was questioned, because the difference between active drugs and placebo was small and the rate of side-effects increased in patients receiving active pharmacological treatment [7]. As non-pharmacological methods, somatic afferent stimuli such as acupuncture [8] and transcutaneous electrical nerve stimulation [9] have been used clinically for the treatment of OAB. However, the lack of appropriate control stimuli makes it difficult to determine whether the effect of somatic afferent stimulation is independent of the placebo effect [8]. It has been reported that not only electrical stimulation but also a variety of noxious somatosensory stimuli, particularly applied to the perineal area, consistently produced a decrease in the frequency of micturition contractions in anesthetized animals [10–13]. In the case of non-noxious mechanical stimuli, we have recently found that the efficacy in suppressing micturition reflex depends on the materials in contact with the skin in anesthetized rats [14, 15]. When gentle stimuli were applied for 1 min by slowly rolling an elastomer roller on top of the perineal skin, both micturition contractions and pelvic efferent nerve discharges were suppressed during and after stimulation [14]. If similar effects can be seen in humans, it may be useful as a self-administered therapy for nocturia. On the contrary, when we used a hard plastic roller, the same gentle stimuli applied to the same skin area did not produce significant inhibition [15]. The two stimuli applied with different rollers, being difficult to distinguish by our senses, would be useful in excluding the placebo effect in a clinical study.

The aim of this study was to examine the efficacy of gentle perineal stimuli, applied by participants at bedtime, to reduce the frequency of nighttime urination in community-dwelling elderly women. For this purpose, we performed a cross-over, placebo-controlled, double-blind randomized clinical study using two different rollers, which were shown to have a different impact on micturition contractions in recent animal studies [14, 15]. Because somatic afferent stimuli have been used for the treatment of OAB [8, 9], we hypothesized that perineal stimulation would be effective for nocturia in elderly women, particularly those with OAB. However, when considering clinical relevance, we performed the minimum duration of intervention on elderly women having nocturia, regardless of the presence or absence of OAB, and accordingly carried out the analyses.

## Methods

The protocol for this clinical trial and supporting CONSORT checklist are available as supporting information; see S1 and S2 Protocols and S1 CONSORT Checklist. The present study was conducted at Tokyo Metropolitan Geriatric Hospital and Institute of Gerontology between January and March 2014 in Tokyo. The study was conducted in accordance with the Declaration of Helsinki and approved by the Human Research Ethics Committee of the Tokyo Metropolitan Geriatric Hospital and Institute of Gerontology. We have changed the protocol from the ethics approval document to exclude men, decrease the numbers of participants, and change the method of analysis for the primary outcome. Written informed consent was obtained from all participants. This trial was registered in the University Hospital Medical Information Network Clinical Trial Registry (UMIN-CTR [UMIN000015809]) on December 1, 2014 (http://www.umin.ac.jp/ctr/index-j.htmhttp://www.umin.ac.jp/ctr/index-j.htm). This study was rapidly undertaken due to the fact that access to participant cohorts was permitted at the same time that the time limit for expenditure of the research funds was approaching. Following permission, to conduct the trial, ethical approval was obtained; however, registration to UMIN-CTR could not be achieved by the time of recruitment and randomization of participants. We noticed after the study had been completed that UMIN-CTR accepted trial registration even after participant randomization [ctr_faq_e1http://www.umin.ac.jp/ctr/UMIN-CTR_e_FAQ.htm#ctr_faq_e1; under 2)-7; accessed on March 30, 2015]. Hence, we have agreed with the significance of UMIN-CTR and registered this study. Such late registration does not affect study results and participants.

### Participants

**Fig 1:**
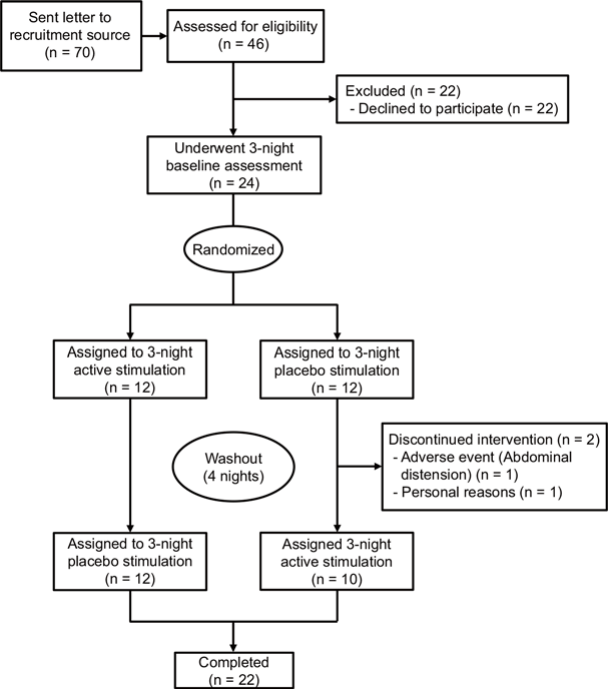
CONSORT flow chart. The flow chart illustrates the basic layout of the clinical trial including steps involved in recruitment of study participants and the actual steps in the clinical trial.

Participants were screened using surveys based on geriatric syndromes, including urinary incontinence, conducted at the Tokyo Metropolitan Institute of Gerontology. One thousand and sixteen elderly women were chosen randomly from the Basic Resident Register of Itabashi-ku in Tokyo [16, 17]. Out of 669 women who participated in the survey, we mailed a recruitment survey to 70 potential participants who had described urinating ≥2 times at night and asked them to participate in a face-to-face interview. A response was obtained from 46 women, of whom 24 were eligible and then enrolled (Fig 1). The inclusion criteria included three main points: 1) having nocturnal urination ≥2 times per night as assessed by a questionnaire; 2) being able to visit our hospital during study periods; and 3) willing to participate after explanation of our study protocols. The exclusion criteria included: underlying diseases and conditions that should be excluded when diagnosing OAB such as bladder cancer, interstitial cysts, urogenital infections [18], or being unsuitable as a participant due to a cognitive disorder. For the diagnosis of underlying diseases and conditions to be excluded, medical histories were obtained, and bladder ultrasonographs and urine laboratory analyses were performed by a urologist. There was no systematic bias in the eventually enrolled sample. The presence or absence of OAB was evaluated using an OAB symptom score, which was based on four symptoms: 1) daytime urination frequency; 2) nighttime urination frequency; 3) urgency; and 4) urgency incontinence during a 1-month recall period [1, 18].

### Study design and schedule

The present study was designed as a randomized, placebo-controlled, double-blind cross-over trial using two different rollers having a different impact on micturition contractions as was shown in recent animal studies [14, 15]. We set a minimum duration for the study period, including a 3-day stimulation period and a 4-day recovery period, based on results from a preliminary study in four OAB patients (unpublished material). The volume and frequency of nighttime urination was assessed by frequency volume charts maintained for 3 days as the baseline assessment period. After that period, participants received either an active or placebo stimuli roller. The stimulation period lasted for 3 days. After a 4-day resting period, participants performed the other stimuli for an additional 3 days. On the day after the each 3-night stimulation period had ended, researchers visited the participant’s home to collect completed frequency charts and the used roller and to provide a new frequency chart and roller (first visit only). For safety assessments, the presence or absence of adverse events were determined by the researcher at this visit and recorded on the charts. The study period lasted 15 days for each participant.

### Skin stimulation

**Fig 2:**
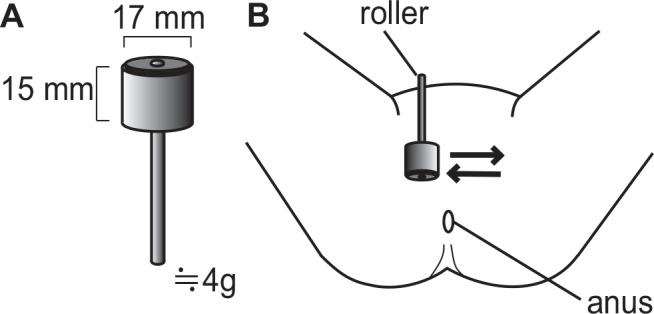
A method for a perineal skin stimulation. (A) is a specification of roller, (B) is stimulation site.

We used two different rollers, each having the same size of 17 mm in diameter and 15 mm in length and weighing 4 g (Fig 2A). These rollers have a similar appearance (same shape and similar color). The only difference between the rollers was surface material that would come into contact with the skin. A roller (SOMAPLANE, Toyoresin Co., Shizuoka, Japan) having a smooth and soft elastic surface (made of elastomer) as described [14] was used for the active stimulation. A roller having a hard non-elastic surface (made of polystyrene, custom-made for our research use by Toyoresin Co.) was used as the placebo, since stimuli with a hard surface did not influence micturition contractions in anesthetized rats [15]. We have confirmed in healthy volunteers [19] that it was possible to camouflage the two types of rollers, by asking whether the roller was active or placebo and by comparing answers between two types of rollers. However, in the present study, we did not confirm this.

Each participant applied stimuli with the roller to their perineum (Fig 2B). We instructed each participant on how to apply the stimuli once-a-day before bedtime for 3 consecutive days. This was done by gently and slowly rolling the roller over top of skin for a period of 1 min, using a force equivalent to the roller’s weight of 4 g. The “dose” of stimulation was determined by the preliminary study performed on OAB patients (unpublished), using the active roller. The participants were not told about the firmness of application of the stimulus. We confirmed that participants properly performed skin stimulation by two parameters: (1) participants completed time of skin stimulation on the frequency chart and (2) researchers visited participant’s home the morning after the 3-night stimulation period had ended and asked them about their skin stimulation performance and any difficulties associated with the study.

After each session ended, the participants were asked their perception on the simplicity of roller use by a written questionnaire with a five-point scale: 1) very difficult; 2) difficult; 3) no opinion; 4) simple; and 5) very simple.

### Outcome measure

The primary outcome was the mean change in nighttime frequency of micturition as determined by a 3-day frequency chart, maintained only at night. We defined nighttime as the period between bedtime (participants intended to sleep) and arising (participants initiated daily activities). Participants completed the times of each micturition during the nighttime period. Mean nocturnal micturition frequency for 3 nights (times/night) was calculated for each baseline, and active and placebo stimulation periods. In two cases participants missed entering data into the frequency chart on one night; mean micturition frequency was obtained from the data of two nights. There were no missing data on frequency charts from the other participants. To focus on frequency during the stimuli periods and to reduce the participants' workload, urine volume measurements were performed only at baseline.

### Sample size

The sample size was based on the preliminary estimates from the study results indicating that nocturnal micturitions ≥2 voids per night may be associated with impaired QOL [36]. To detect a clinically meaningful difference in the frequency of urination of one void per night in mean change from baseline between active and placebo treatments (standard deviation was assumed to be 0.9 [20]) with 90% power and a two-sided significance level of 5%, the required group size was 11 participants with OAB. Forty-nine percent of nocturia occurring in elderly patients (60–80 years old) was related to OAB [21]. Hence, the total sample size required was 23 participants.

### Randomization

The 24 participants were divided into two groups with an allocation ratio of 1:1, according to computer-generated random numbers at the Teikyo Heisei University by S.M. Allocation of which group would apply the active roller for the first three days was also randomly generated.

### Blinded study

The rollers were placed in similar cartridges before use. Allocation and packaging were blinded except to one of the researchers who did not have any contact with the participants. The other researchers were not allowed to open the cartridge, to view, or to touch the surface of the roller. Participants were not informed as to which type of roller was expected to be effective.

### Statistical analysis

The change in micturition frequency during the stimulation periods from that seen in the baseline period was calculated. Additionally, a difference in micturition frequency between the active and placebo stimuli periods was computed. These values were compared using a paired or unpaired t-test (two-tailed), after confirming normal distribution of the data using the Shapiro–Wilk normality test (Prism 6 software, GraphPad Software, La Jolla, CA, USA). The effect size (d) was estimated [22]. A correlation between a difference in micturition frequency (active-placebo) and each variable was evaluated as bivariate analysis with the Spearman’s rank correlation coefficient (two-tailed). In addition, multiple regression analysis was used as multivariate analysis to explore how explanatory variables affect the difference in micturition frequency (IBM SPSS Statistics version 19.0.0, Tokyo, Japan). Two variables, i.e., baseline frequency or OAB symptom score, entered for the regression analysis. Participant’s perception on simplicity of roller use was statistically analyzed by Chi-square test. The statistical significance level was set at 5%. Data were expressed as mean ± standard deviation.

## Results

The flow chart for the layout and steps in the study is presented in Fig 1. Of the 24 participants enrolled, 22 completed all study sessions and were included in all analyses. Two participants discontinued the treatment before completion of the study. One was due to an adverse event consisting of a feeling of abdominal distension on the second and first day of placebo and active stimulation period, respectively. The other declined to participate after placebo stimuli period due to personal reasons not related to the study. No adverse events were reported during either active or placebo stimuli periods by the other 22 participants.

### Participant characteristics

The age of the 22 participants ranged between 79 and 89 years old. The presence of OAB, evaluated by the OAB symptom score, was nine out of the 22 (40.9%). Baseline characteristics of the nine OAB and 13 non-OAB participants were shown in Table 1. There were non-OAB participants with residual volumes >100 ml, who were receiving anticholinergic drugs and who suffered from Parkinson’s disease and diabetes. These symptoms are well known to affect bladder functions but were not observed in OAB participants. Other geriatric symptoms such as hypertension, hyperlipidemia, and osteoporosis were observed in both OAB and non-OAB participants. Of the nine participants with OAB, five performed the active stimuli session first and four did the placebo stimuli session first.

**Table 1:** Baseline characteristics.

	OAB (n = 9)	non-OAB (n = 13)
Age (years old)	82.33 ± 1.66	83.08 ± 3.12
Urination frequency (times/night)	3.52 ± 1.43	2.97 ± 0.99
Volume per void (mL)	234.8 ± 87.9	233.4 ± 64.3
Residual volume >100 ml	0	4
Anticholinergic drugs	0	2
Parkinson’s disease	0	1
Diabetes	0	3
Hypertension	4	6
Hyperlipidemia	1	2
Osteoporosis	2	6

Data are expressed as mean ± standard deviation.

### Frequency of nocturnal urination

**Fig 3:**
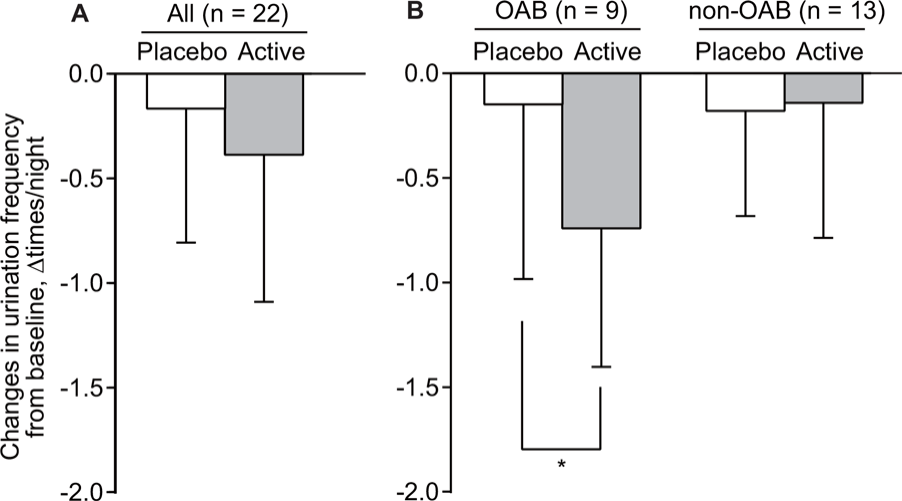
Changes in nighttime urination frequency during placebo and active stimulation periods from that during the baseline assessment period. Data are expressed as mean ± standard deviation. *p < 0.05; tested by paired t-test between active and placebo stimulation periods.

The frequency of urination in all 22 participants during the baseline period was 3.2 ± 1.2 times/night. Changes in nighttime urination frequency during active stimulation from that during baseline period was greater than that during placebo stimuli period, but the difference was not statistically significant (−0.39 ± 0.7 vs. −0.17 ± 0.6, 95% CI for stimulation difference -0.067 to 0.51, p = 0.13, d = 0.46; Fig 3A).

Subsequently, further analyses were performed by dividing participants into groups with or without OAB. In the nine participants with OAB, a change in nighttime urination frequency during the active stimulation period from that seen during the baseline period was significantly greater than that during the placebo stimuli period (−0.74 ± 0.7 vs. −0.15 ± 0.8, 95% CI −1.1 to −0.07, p = 0.031, d = 1.12; Fig 3B left). In contrast, in participants without OAB, changes in micturition frequency during the active and placebo stimuli periods were not significantly different (−0.14 ± 0.6 vs. −0.18 ± 0.5, 95% CI −0.33 to 0.26, p = 0.78, d = 0.09; Fig 3B right).

**Fig 4:**
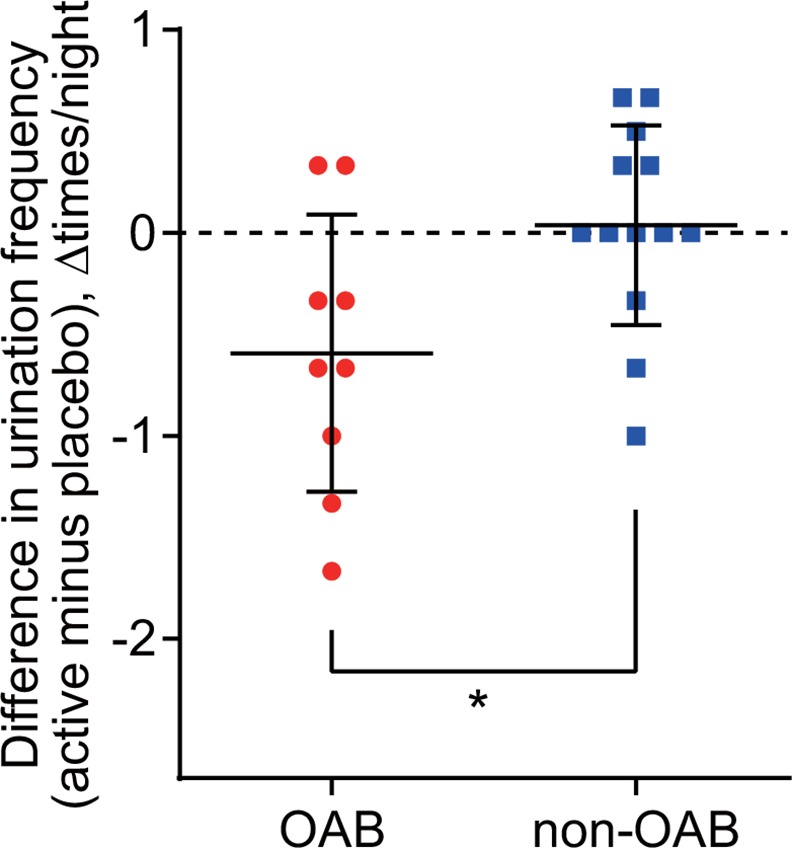
Differences in frequency of nighttime urination between active and placebo stimuli. Data are expressed as mean ± standard deviation. Each dot represents individual data. *p < 0.05; tested by unpaired t-test.

Fig 4 shows a summary of the degree of differences in frequency of nighttime urination between the active and placebo stimuli periods for each participant. To exclude the placebo effect, values of nighttime urination frequency during the placebo stimuli period were subtracted from those during the active stimuli period. The unpaired t-test revealed that the frequency difference was significantly greater in participants with OAB, compared to those without OAB (−0.59 ± 0.7 vs. 0.04 ± 0.5, 95% CI for group difference −1.2 to −0.11, *p* = 0.02, d = 0.92).

To clarify factors that are associated with a change in nighttime urination frequency during the active stimuli period, bivariate analysis was performed. The differences in urination frequency between active and placebo stimuli showed no significant correlation with urination frequency during the baseline period (Table 2). On the other hand, the frequency difference was negatively correlated with the OAB symptom score (r = −0.57, p = 0.0059). Similar results were also obtained by multivariate analysis (Table 2).

**Table 2:** Association between difference in urination frequency and explanatory variables (n = 22).

	Bivariate Analysis		Multivariate Analysis	
Explanatory variables	Correlation Coefficient	P-value	Standardized Partial Regression Coefficient^‡^	P-value
Urination frequency	-0.13	0.56	-0.04	0.84
OAB symptom score^†^	-0.57	<0.01	-0.69	<0.01
adjusted R^2^	―		0.45	

^†^Overactive bladder (OAB) symptom score; Total score can therefore range from 0 to 15, with higher scores indicating increasing symptom severity.

^‡^Variance inflation factors < 10.

### Perception on simplicity of skin stimulation method

The majority of participants answered that they felt it was simple to use the rollers (Table 3). The Chi-square test revealed that there was no significant difference in perception scores between active and placebo rollers (*p* = 0.45).

**Table 3:** Participant’s perception on simplicity of roller use.

	Active roller	Placebo roller
Very difficult	1	2
Difficult	1	3
No opinion	7	4
Simple	10	7
Very simple	3	6

## Discussion

The present study examined the effects of gentle perineal stimuli on the frequency of nighttime urination in elderly women with nocturia. Experiments were performed in a randomized, placebo-controlled, double-blind crossover manner by using two different rollers with different surface materials (soft vs. hard). Participants applied the perineal stimuli for 3 consecutive days for a period of 1 min at bedtime. The results showed that stimulation with a soft elastomer roller (used as the active roller), but not with a hard polystyrene roller (used as the placebo), decreased frequency of nighttime urination, especially in participants with OAB. The inhibitory effect on the frequency of urination by the active stimulation was consistent with the inhibitory effect on the frequency of rhythmic micturition contractions of the expanded bladder by the same stimuli in our previous animal studies under anesthesia [14, 15]. The difference in the inhibitory efficacy between stimulation with the two different rollers was also in accordance with our animal study [15]. The present results suggest that an inhibitory effect on bladder contraction caused by OAB can be achieved by perineal stimulation.

### OAB vs. non-OAB

In contrast to the significant decrease in the frequency of nighttime urination by active stimulation in participants with OAB, there was no significant effect on the frequency of urination in participants without OAB. That was predicted and then confirmed by our present results. In particular, a greater reduction in the frequency of nighttime urination was seen in participants with higher OAB symptom scores, suggesting that the active roller is effective for inhibiting bladder contractions associated with OAB. However, since the pathogenesis of OAB has not been thoroughly clarified, we cannot explain the cause(s) of the different efficacy of the active stimuli on OAB vs. non-OAB. A variety of factors known to affect bladder functions (such as residual volume, use of anticholinergic drugs, Parkinson’s disease, and diabetes) appeared to be involved as causes of nocturia in the present non-OAB participants. Although we did not examine polyuria or nocturnal polyuria in the present study, these two factors could be the main cause of nocturia in non-OAB participants. In future studies examining long-term stimulation, we should just focus on nocturia with OAB.

### Efficacy

The effect of the active stimuli was significant in nocturia participants with OAB and the decrease of frequency of nighttime urination during active stimuli was about 0.59 times/night compared with that during placebo stimuli. This effect was significantly greater than that seen with drugs that are highly recommended for OAB, such as tolterodine or solifenacin, which are administered for 4–12 weeks. Although current treatments for OAB are recommended in various guidelines, based on the statistically significant differences compared to placebo, the clinical relevance of this effect [23–25] is very small. Such drug treatment for OAB is not often directed at nocturia, but the recent trial concerning the effect of fesoterodine on nocturnal urgency as the primary outcome also showed only a small decrease (by 0.21 times/night) when compared to placebo [26]. Therefore, the present results suggest that gentle perineal stimulation with the active roller can be useful as an alternative for pharmacological nocturia treatments. In the case of non-pharmacological therapy such as acupuncture [27], moxibustion [28], transcutaneous electrical nerve stimulation [29], and magnetic stimulation [30], 1–12 weeks of treatment decreased the frequency of nighttime urination by 0.3–2.4 times/night when compared with baseline levels. Such somatosensory stimuli were not always possible to administer in a randomized clinical trial [8]. However, the present results may partially explain the efficacy of these alternate treatments.

### Safety

During the course of the study, adverse events were not reported except for one participant who discontinued because of the feeling of abdominal distension. That adverse event occurred during both the active and placebo stimuli periods. Adverse events selectively produced by active stimulation were not observed in this study. Safety of the active stimulation was predicted because of very gentle skin stimulation and then confirmed by the present results. The dropout rate after randomization was only 8% even though the ages of the participants were quite high (79–89 years old), supporting the safety of self-administered cutaneous stimulation. However, safety should be carefully analyzed in trials carried out over a longer period.

### Placebo stimulation vs. active stimulation

In contrast to the decrease in nocturnal frequency of urination seen with the active roller, placebo stimulation did not produce any consistent effect on urination frequency. Therefore, the placebo effect was negligible in this study. We used two different rollers for stimulation. The difference between the two sets of stimuli was produced solely by the difference in the roller surface materials in contact with skin. Both stimuli were gentle and the participant’s perception of the simplicity of roller use was not significantly different (Table 2). Although it is hard to perceptually distinguish between these rollers, the properties of the materials used for the roller surfaces were different; the active roller was soft and sticky, whereas the placebo roller was hard and slippery. Therefore, such differences may produce different excitation frequencies and patterns of various types of mechanoreceptive afferent fibers that innervate the perineal skin. We previously showed that the active stimulation applied to rats' perineal areas excited low-threshold cutaneous mechanoreceptive Aβ, Aδ, and C fibers at mean frequencies of 2, 3, and 8 Hz, respectively [14]. Studies on the effects of electrical stimulation of cutaneous afferent nerves have suggested that inhibitory effects on bladder contractions are dependent on the types of afferent nerve fibers and frequency of evoked discharges [31, 32]. Therefore, it will be important to compare responses of each cutaneous afferent nerve fiber after active and placebo stimuli in the future to clarify the afferent mechanism for inhibition of frequency of nighttime urination.

### Possible mechanisms

The present results, in conjunction with previous animal studies [14, 15], suggest that there are common mechanisms in rats and humans that indicate some form of somatosensory stimuli that inhibit transmissions of micturition reflex pathways. Similarities between the effects of gentle skin stimulation on nociceptive stimulation-induced cardiovascular reflexes in conscious humans and anesthetized rats have also been reported recently [33–35]. In our experiments with anesthetized rats, we recorded discharges from the vesical pelvic efferent nerve fibers or skin afferent nerve fibers and bladder contractions before and after administration of an opioid receptor antagonist. Using these techniques, we showed that excitation of low threshold cutaneous mechanoreceptive myelinated and unmyelinated fibers inhibited the vesico-pelvic parasympathetic reflex; this occurred via activation of the opioid system in the spinal cord and by reducing both the ascending (afferent) and descending (efferent) transmissions between bladder and pontine micturition center [36, 37]. Such inhibitory mechanisms could modify urgency in OAB with or without detrusor overactivity. The mechanism, as revealed by animal experiments, may explain the present clinical outcome of gentle perineal stimulation to reduce the frequency of nighttime urination due to OAB.

### Limitations and future study needed

The major limitation of this study was the restricted treatment period. Nocturia can lead to sleep impairment and lower QOL [38]. Therefore, the reduction in frequency of nighttime urination observed in the present study could lead to improvements in sleep quality and QOL. However, the present stimulation period of 3 days was too short to convincingly assess QOL. Results from neural mechanisms derived from animal studies support the idea that stimulation possibly increases functional bladder capacity; consequently, repeated stimulation for longer periods may gradually induce greater clinical outcome. Further study is needed to examine the effects of repeated stimulation for longer periods, in which the effects on sleep quality and QOL (such as using a Nocturnal QOL Questionnaire [39, 40]) should be examined concurrently.

## Conclusion

The present results provide the first clinical evidence that stimulation of the perineal skin with a soft roller may be effective for OAB-associated nocturia in elderly patients. The significant decrease in the frequency of nighttime urination was obtained by self-stimulation for only 1 min at bedtime once-a-day. Gentle perineal stimulation is expected to be a novel self-administered therapy for nocturia.

## Supporting Information

S1 CONSORT Checklist (DOC)

S1 Protocol Trial Protocol. Approved ethics application form for the present study. This document includes inclusion and exclusion criteria and trial protocol.
(DOCX)

S2 Protocol Trial Protocol written in Japanese. Approved ethics application form written in Japanese (original document) for the present study.
(DOC)
