# Supplementary material for: Effects of a Gentle, Self-Administered Stimulation of Perineal Skin for Nocturia in Elderly Women: A Randomized, Placebo-Controlled, Double-Blind Crossover Trial
Source: PLoS One. 2016 Mar 22;11(3):e0151726. doi: 10.1371/journal.pone.0151726 (PMC4803221; doi:10.1371/journal.pone.0151726)
Supplement: S1 Protocol — Approved ethics application form for the present study. This document includes inclusion and exclusion criteria and trial protocol. (DOCX) [file pone.0151726.s002.docx]

Form #1

| Receipt No. |  |
| --- | --- |

Application form of the Human Research Ethics Committee of the Tokyo Metropolitan Geriatric Hospital and Institute of Gerontology

To the CEO of the center

Date of submission: December, 4^th^ 2013

Affiliation of applicant: Department of urology

Name of applicant: Yutaka Kasuya Seal

|  | | Supervisor’s seal |  |
| --- | --- | --- | --- |
| 1. Review category | Medical research　　　2. ( ) research　　　3. Others | | |
| 2. Title of research | A double-blind randomized cross-over trial for a study on the effectiveness of gentle mechanical skin stimulation on nocturia | | |
| 3. Principal investigator | Department of Urology, Tokyo Metropolitan Geriatric Hospital; Yutaka Kasuya | | |
| 4. Research period | From the date of ethics committee approval to March, 31^st^ 2015 | | |
| 5. Outline of research (please briefly describe the contents of research and provide the name of funding body if any external funds)  (1) Co-researchers: Department of Urology, Tokyo Metropolitan Geriatric Hospital; Koichi Masunaga (representative), Takuji Nagata  Research collaborators: Tokyo Metropolitan Institute of Gerontology; Harumi Hotta, Nobuhiro Watanabe, Hunkyung Kim  : Teikyo Heisei University; Shogo Miyazaki, Kaori Iimura  (2) Purpose: To examine the efficacy of mechanical stimulation of the perineal skin for treating nocturia  (3) Content of research:  We will perform a double-blind randomized cross-over trial using two different gentle tactile stimulation techniques (expected to be effective or not) for nocturia. | | | |

　Please attach one copy of questionnaires and consent form if any.

　Please attach one copy of article in the case that review category is “journal article etc.”

| 6. Research participants (age, gender, community-/facility-dwelling persons, healthy people, honorarium)  Inclusion criteria  1) Both genders (50 or over 50 years old)  2) Urinating more than two times at night (from bedtime to awakening in the next morning)  3) Diagnosed as “overactive bladder” (* refer to “algorism of medical practice for nocturia”)  4) Not medicated for nocturia more than 4 weeks prior to registration  5) Being able to visit our hospital during observation periods (except hospitalized patients)  6) Being able to obtain a written consent for their participation in the present study  Exclusion criteria  1) Visiting clinics/hospitals due to primary illness (diabetes, hypertension etc.)  2) Individuals judged by a physician to be unsuitable as a participant (e.g., cognitive disorder)  No honorarium is provided to participants. |
| --- |
| 7. Research methods (postal mail, face-to-face interview, examination, sample collection, the number of tests, person in charge)  Stim. A or B  Pre-registration  test  3 days  1 day  3 days  1 day  1 day  3 days  3 days  Informed consent  Registration  Stim. A or B  Finish  Resting period  Setting: Examination period is approximately two weeks (see figure above). During this period, participants will visit our hospital/institute for informed consent, pre-registration test, and examinations at the end of observation and follow-up periods. Research collaborators will visit participant’s home to collect salivary samples (samples will be collected four times in total; before and after each stimulation period).  Participants: The expected number of participants is more than 30 in each group (more than 60 participants in total).  Intervention: Gentle tactile stimulation will be applied to perineal skin by participants (self-care) using a device made of elastomer (Somaplane, Toyoresin Co., Shizuoka) or of polystyrene (Toyoresin). The stimulation will be applied with approximate weight of the roller and the velocity of 1cm/sec for 60 seconds. That stimulation is determined as one dose and applied once a day before bedtime.  Primary outcome: Primary outcome is the ratio of participants whose frequency of urination per night (using frequency chart) decreases to a half. The ratio will be compared with that of control group to determine whether they are significantly different.  Secondary outcomes: Overactive bladder symptom score, N-QOL, The frequency of nighttime voiding, duration between bedtime and the first voiding, and hormones in salivary etc. |

| 8. Ethical considerations  　(1) Considerations for protecting human rights of individual participant (privacy, distress/risk)  We will carefully store participant’s personal information obtained through the present study in our institute in order to safely manage and store them. Under the condition that participant’s privacy is protected, only the principal investigator, the co-researchers, the collaborators, the ethics committee of our institute, and domestic and international regulatory agencies can peruse the information. Additionally, even when obtained information is published such as scientific articles, participant’s privacy is protected. Participants who put their signature to consent form are regarded as agreeing with these considerations.  　(2) Means to obtain participant’s consent (the participant themselves, any cases that need to obtain other’s agreement)  The principal investigator, the co-researchers, and the collaborators will sufficiently explain the details of the research prior to their participation based on the participant information sheet and the consent form. The principal investigator and the co-researchers will obtain participant’s voluntary consent in written form, after providing enough time for consideration of their participation or withdrawal.  　(3) Potential participant’s disadvantage and risks and scientific advantage  This research will be supported by the translational research fund of Tokyo Metropolitan Institute of Gerontology and the fairness of the interest is maintained.  　 (4) Insurance to indemnify health damage associated with clinical study and the details of other necessary practice and informed consent  In the case that health damages such as serious side effect are noticed during or after the course of the present study due to participation in the present study, appropriate medical treatment, which is the same as the regular treatment, is provided. Medical costs associated with the treatment are covered by conventional health insurance and no compensation by money will not be provided. Participants who have provided their signature on consent form are regarded as agreeing with abovementioned considerations. |
| --- |
| 9. Others |
