# Supplementary material for: Effects of a Gentle, Self-Administered Stimulation of Perineal Skin for Nocturia in Elderly Women: A Randomized, Placebo-Controlled, Double-Blind Crossover Trial
Source: PLoS One. 2016 Mar 22;11(3):e0151726. doi: 10.1371/journal.pone.0151726 (PMC4803221; doi:10.1371/journal.pone.0151726)
Supplement: S2 Protocol — Approved ethics application form written in Japanese (original document) for the present study. (DOC) [file pone.0151726.s003.doc]

様式１号

| 受付番号 |  |
| --- | --- |

　　　　　　　東京都健康長寿医療センター倫理審査申請書

センター理事長　　　殿　　　　　　　　　　　　　　　　　　　２０１３年１２月４日

　　　　　　　　　　　　　　　　　　　　　　　申請者　所属・職　泌尿器科・

　　　　　　　　　　　　　　　　　　　　　　　　　　氏　名　　　粕谷 豊 　　　印

|  | | 所属長の印 |  |
| --- | --- | --- | --- |
| １　審査対象 | 医学的研究　　　２（　　　　）研究　　　３　その他 | | |
| ２　研究課題名 | 夜間頻尿に対する軽い機械的な皮膚刺激の  有効性に関する二重盲検ランダム化交叉群間比較試験 | | |
| ３　研究責任者 | 東京都健康長寿医療センター 泌尿器科　粕谷 豊 | | |
| ４　研究期間 | 倫理委員会承認後　～２０１５年３月３１日 | | |
| ５　研究の概要※（研究内容は簡潔に、外部資金を予定している場合は資金元を明記。）  　　（１）研究分担者：東京都健康長寿医療センター 泌尿器科　桝永 浩一（代表）  東京都健康長寿医療センター 泌尿器科　永田　卓士  研究協力者：東京都健康長寿医療センター 研究所　堀田 晴美、渡辺　信博、金　憲経  帝京平成大学　宮崎 彰吾、飯村　佳織  　　（２）目的：会陰部への機械的な皮膚刺激が夜間頻尿に有効であるか検討する。  　　（３）研究内容：夜間頻尿に対して、会陰を効果が期待できる機械的（軽い触圧）に刺激する方法と効果が期待できない機械刺激方法を無作為に割付け、夜間頻尿に対する有効性を二重盲検ランダム化交叉群間比較試験によって評価する。 | | | |

　調査票、承諾書がある場合は、写しを１部添付して下さい。

　審査対象が「論文等」の場合は、その写しを１部添付して下さい。

| ６　研究対象（年齢、性、地域住民・施設居住者、健常者、謝礼の有無）  被験者の適格基準  1) 50歳以上の男女  2) 夜寝てから朝起きるまでに、1日2回以上トイレに起きる方  3) 「過活動膀胱」と診断された方（※別紙「夜間診療の診療アルゴリズム」を参照）  4) 夜間頻尿に対して医師から処方された経口薬を登録前に4週間以上継続投与していない方  5) 観察期間内に当院に通院できる方（入院患者は除外）  6) 本試験の参加に関して同意が文書で得られる方  被験者の除外基準  1) 基礎疾患（糖尿病、高血圧など）で通院している方  2) その他、医師の判断により対象として不適当であると判断された方（例：認知機能障害）  被験者への負担軽減費（謝金）は、なしとする。 |
| --- |
| ７　研究方法等（郵送、面接、テスト、検体の採取、回数、直接の担当者）  同意取得  登録前検査  登録  3日間  1日間  1日間  刺激AまたはB  3日間  休養期間  3日間  刺激AまたはB  3日間  終了  1日間  セッティング：試験期間は、およそ2週間（上図参照）、うち同意取得、登録前検査、観察期間終了時、追跡期間終了時の2回通院にて調査。唾液試料（各介入期間の前後計4回採取）は研究協力者が被験者の自宅を訪問し、回収。  参加者：予定症例数は各群30例以上（計60例以上）  介入：被験者自身（セルフケア）により、エラストマー製の器具（Somaplane, Toyoresin Co.Shizuoka）またはポリスチレン製の器具（Toyoresin）を用いて、軽い触圧（自重程度の圧力）でかつ、1cm/秒の速度で60秒間、会陰部の皮膚を刺激する。以上を1回量とし、1日1回就寝前に行う。  主要評価項目：主要評価項目は夜間排尿回数（排尿日誌による）が半減した割合とし、対照群と比較して有意差があるか検討する。  副次的評価項目：過活動膀胱症状質問票、N-QOL、夜間排尿回数、就眠後、第一排尿までの時間および唾液中に含まれるホルモンなど。 |

| ８　研究における倫理上の配慮  　⑴　対象となる個人の人権の擁護のための配慮（プライバシー、苦痛・危険性）  本研究で得た被験者の個人情報が安全に管理・保管できるよう当院内にて厳重に保管する。被験者の秘密が保全されることを条件に試験責任者、試験分担者、試験協力者、当院倫理審査委員会、国内外の規制当局のみが閲覧できることとする。また、得た情報を学術論文等で公表する場合であっても、被験者の秘密は保全される。以上について、被験者は同意書に署名することにより同意したこととする。  　⑵　対象者の同意を得る方法（対象者本人、対象者以外の同意を要する場合）  研究責任者、研究分担者、研究協力者は被験者に対して別に定める説明・同意文書に基づき、本研究に参加する前に研究の内容について十分に説明する。当該者に本研究に参加するかどうかについて十分考える時間を与えた後、研究責任者、研究分担者は当該者本人の自由意思による同意を文書で得る。  　⑶　研究により生じる対象者への不利益及び危険性と研究上の利益の予測  本研究計画は、東京都健康長寿医療センター研究所・トランスレーショナルリサーチ基金により行われる予定であり、利害関係についての公正性を保っている。  　（4）臨床研究の実施に伴う被験者に生じた健康被害の補償のための保険その他必要　な措置の内容及びインフォームド・コンセント  本研究の参加または終了後に本研究に参加したことが原因となって、重篤な副作用などの健康被害を受けた場合には、通常の診療と同様に適切に治療を行う。その際の医療費は通常の保険診療にて賄い、金銭での補償金の支払いはない。以上について、被験者は同意書に署名することにより同意したこととする。 |
| --- |
| ９　その他 |
